# From basic social neurobiology to better understanding of neurodevelopmental disorders

**DOI:** 10.1111/gbb.12818

**Published:** 2022-06-10

**Authors:** Andrey E. Ryabinin

**Affiliations:** ^1^ Department of Behavioral Neuroscience Oregon Health & Science University Portland Oregon USA

This volume concludes our three‐part series of Genes, Brain and Behaviors special issues. The previous two volumes started with research on autism and neurodevelopmental disorders, concluding that their full understanding is impossible without understanding of innate social behaviors, and continued into research on mechanisms of such innate behaviors across several animal species.^1^, ^2^ The current special issue continues the analysis of innate social behaviors and then brings us back to studies on animal models of neurodevelopmental disorders, hopefully with a greater appreciation for intricacies of social neurobiology.

In their research paper, Goncalves, Kareklas and colleagues have elegantly addressed a fundamental question on evolution: whether the motivational and the cognitive components comprising social behavior evolved independently or through selective pressure to increase sociality.^3^ To address this question, the authors phenotypically characterized several lines of male and female zebrafish (*Danio rerio*) in four behavioral tests: shoal preference, conspecific recognition, object recognition and open‐field. This characterization clustered behaviors across three principal factors: motivation, cognition and anxiety. Importantly, the social tendency module in this analysis aligned with object and social exploration, and clustered separately from object discrimination and social discrimination, which aligned together. These data provide strong support for the hypothesis that social recognition and social motivation did not evolve together as a common phenotype and instead separately co‐opted general cognitive and motivational mechanisms. This conclusion is further supported by analysis of single nucleotide polymorphisms (SNPs) and behavioral modules. The genetic analysis shows that SNPs in candidate genes associated with social behaviors are statistically associated with the motivational but not the cognitive module. This finding is in agreement with the recent report showing that zebrafish lacking functional oxytocin receptors are deficient in social and object recognition but have intact social motivation.^4^ While the well‐characterized behavioral repertoire and genetics of zebrafish allows the researchers to conclude that social recognition and social motivation can function as independent entities, this conclusion amplifies the need to study neural mechanisms regulating specific social behaviors, rather than the more generalized “social brain.”

Not all specific social behaviors, including social behaviors characteristic of humans, can be studied in standard laboratory species. The previous volume of the special issue highlighted the importance of choosing appropriate species for such studies by discussing the power of experiments in socially monogamous prairie voles for understanding the neurobiology of pair‐bonding.^5^, ^6^ The research paper by Mederos, Duarte and colleagues in the current issue initiates the development of a novel fish model that could enhance our understanding of pair‐bonding.^7^ The paper is based on the premise of frequently observed social and genetic monogamy across several related species of seahorses,^8^, ^9^, ^10^ which suggests that they are capable of pair‐bonding. In their paper, the authors investigate color changes as a form of social communication in lined seahorses (*Hippocampus erectus*). They demonstrate that the color change (increase in brightness) occurs more frequently, especially in male fish, when they are paired with their pair mate, than when they are paired with an opposite‐sex stranger. In a subsequent preliminary whole brain RNA sequencing analysis authors detect a substantial overlap in pairing‐induced changes in gene expression between male seahorses and prairie voles. Remarkably, oxytocin receptor is among upregulated genes in paired female seahorses, in agreement with its well‐known importance in pair‐bonding in other species.

Animal species adopt different modes of social communication depending on evolutionary constraints. Ultrasonic vocalizations (USVs) are of great importance for social communication in rodents. Previous research demonstrated that communication via USVs depends on age, sex and can also differ between mouse strains.^11^ However, the detailed understanding of differences in such communication and its relationship to underlying behavioral situation is missing. In their research paper, Caruso et al.^12^ provide an extensive analysis of vocal repertoire across several early postnatal ages and in adults of three mouse strains: the commonly used inbred C57BL/6N, the highly social inbred FVB and the outbred CD‐1. Moreover, the USVs were analyzed across three behavioral situations: social separation in mouse pups, adult male–female encounters and adult female–female encounters. In parallel, the authors assess the behavioral repertoire of adult mice during the interactions. While the USV repertoire of mice changes from early postnatal to adult age, strain‐ and social context‐dependent differences are ever present in all assessments of vocalizations.

The behavioral repertoire of animals can not only differ between different ages, genotypes and social context, but can be even different between cagemate rodents of the same strain, sex and age. Animals living in groups usually organize themselves into social hierarchies. Behavioral repertoires, as well as general well‐being, are different between socially‐dominant and subordinate individuals within these groups.^13^ Park and colleagues in their research paper address an intriguing question whether social rank affects empathy‐like behavior in mice.^14^ The authors determine social ranks in male C57BL/6J mice using the well‐established tube test for social ranking and then evaluate these mice for their ability to develop empathy‐like observational fear learning (OFL). In OFL, “observer” mice acquire fear from “demonstrator” mice undergoing fear conditioning. The authors demonstrate that intermediately ranking observers are better at acquiring fear from dominant than from subordinate demonstrators. In the same direction of effect, subordinate observers are better than dominant cagemates at acquiring fear from intermediately ranking demonstrators. Moreover, the OFL is dependent on the steepness of the social hierarchy. These findings are in agreement with a previous study showing that the subordinate rank in rats enhances social fear transmission,^15^ suggesting generality of this effect across species. However, the previous studies were not able to discern whether this increased fear is due to familiarity of the observer with the social rank of the demonstrator or due to some intrinsic quality of dominant demonstrator. Park and colleagues rule out the second possibility by showing that social ranking does not affect OFL from unfamiliar mice. Their study definitively determines higher empathy‐like responses in more subordinate versus more dominant cagemates, at least in male mice. While not the main focus of the study, this article also shows that both single‐housing and crowding strongly inhibit empathy‐like OFL in mice calling to attention the importance of housing conditions for assessing social behaviors.

The latter observation is well in line with the next article in this special issue. The research paper by Winiarsky, Kondrakiewicz and colleagues compares effects of housing, habituation and testing conditions in males of commonly used C57BL/6J mice versus BTBR T+ Iptr3tf/J mice (BTBR) mice.^16^ The BTBR mice are a recognized idiopathic model of aspects of autism spectrum disorder (ASD), and as such demonstrate deficits in social behaviors.^17^, ^18^ In the first series of experiments, the authors test these mice in the 3‐chamber social affiliation test under several low and high stress conditions. These conditions range from testing non‐habituated mice under relatively bright light conditions to habituated to handing and transportation mice, housed in enriched environment and tested in dim light. Behavior of the mice changes substantially depending on these conditions, at time obscuring the ability to detect strain differences. In the second series of experiment, the authors use an automated Eco‐HAB system,^19^ which allows long‐lasting evaluations of group‐housed mice without disturbing them, to assess differences in social behaviors between these strains of mice. Among the identified striking differences, C57BL/6J mice demonstrate stability of social structure, whereas only limited subgroups of interactive BTBR mice engage in sporadic social interactions in these settings. Authors discuss the pros and cons for each of the two approaches for evaluating differences in the Systems for Social Processes Domain between mouse strains and highlight how differences in experimental conditions can clearly contribute to varied results.

The special issue is concluded with two important reviews on neurodevelopmental disorders and their animal models. The review by Benedetti and colleagues takes on one genetic mechanism with high penetrance for such disorders.^20^ Namely, authors screened over 750 publications and analyzed 58 studies in humans and 16 studies in mice that focused on behavioral characterization of subjects with copy number variations (CNVs) in DNA segments 16p11.2 and 22q11.2. CNVs on these genetic loci are associated with substantially increased risk for ASD, schizophrenia and attention deficit disorder.^21^, ^22^ Mutant mice with deletions of appropriate DNA loci have been developed. These mouse mutants provide models with excellent construct validity for the corresponding CNVs in humans. Authors review deficits in the several social domains in subjects with CMVs (involving social interaction, social communication, social memory and social cognition) and criteria for their identification. They report on several matching changes in tests of sociability, social interaction and social memory and communication (vis USVs) in the mutant mice. These commonalities in observations between human and mouse studies are promising for better understanding of associated disorders. However, on one hand, the number of behavioral tests assessed mice with relevant CNVs are sparse, compared with the vast array of murine social behavioral tests available. On the other hand, even the performed tests do not align well with the psychiatric diagnostic criteria used in humans. While highlighting the deficiencies, Benedetti and colleagues discuss several exciting avenues for further testing in rodent models (for example, assessing recognition of visual cues, parental influences, immunological mechanisms and effects of microbiome).

While an increase in studies using relevant tests on social behaviors would benefit understanding of deficits in social behaviors associated with CNVs, the final paper in this issue questions how the animal models of neurodevelopmental disorders should be optimally utilized. Silverman and colleagues report on recommendations of the workshop held by the Autism Science Foundation with the goal of improving utilization of such models.^23^ The resulting review collected 148 and analyzed 69 articles on ASD mouse models. The analysis reveals inconsistent reporting patterns in behavioral performance, experimental design and insufficient alignment of behavioral testing with diagnostic criteria of ASD. The authors emphasize the need to focus on construct validity of models, rather than on their face validity, as well as the importance of not underestimating the complexity of behavior in animal models. Silverman and colleagues acknowledge the challenges of translating between mouse and human behaviors, the complication of results due to use of various genetic backgrounds, use of different methodologies across labs, problems with inter‐laboratory reproducibility, often insufficiently clear or incorrectly applied statistical analyses, lack of appreciation for temporal trajectories of phenotypes, the need to go beyond mice to represent the full repertoire of complex behaviors and the need to characterize models of neurodevelopmental disorders across multiple behavioral domains. The authors recommend that researchers assess not only social behavior in their models but measure cognitive and motor abilities, assess their models across a spectrum of comorbidities, keep in mind the role of olfaction in rodent social interactions, control for underlying motivation of subjects in their studies, all the while sticking to meticulous reporting of experimental design, detailed methods, materials, results and statistical analyses.

We conclude this series of special issues on social behaviors, autism and neurodevelopmental disorders by emphasizing that this exciting research field exhibits much motivation to go beyond superficial observations of simplistic phenotypes. The front cover image of this special issue, by Dr. Yangmiao Zhang, illustrates that participants in social interactions come with different personal experiences and backgrounds. These experiences guide them to exhibit various behaviors, including social bonding. May the complexity of social behaviors serve as a common challenge to bond neuroscientists in the pursuit of intricate underlying mechanisms.

## Data Availability

Data sharing is not applicable to this article as no new data were created or analyzed in this study.
